# Supplementary material for: Investigating the Use of a Liquid Immunogenic Fiducial Eluter Biomaterial in Cervical Cancer Treatment
Source: Cancers (Basel). 2024 Mar 20;16(6):1212. doi: 10.3390/cancers16061212 (PMC10969426; doi:10.3390/cancers16061212)
Supplement: Supplementary file 1 [file cancers-16-01212-s001.zip › Table S3.pdf]

VRL – Maryland, LLC
401 Professional Drive, Suite 210
Gaithersburg, MD 20879
Phone: 1-800-804-3586

Date: 21 November 2023
Client: Johns Hopkins University
Pathologist: Dr. Dan Ragland

Table S3. Histopathology Report for Female mice (n = 3) Day 1 post-treatment.

Mouse Accession	23025246	23025247	23025248	23025249	23025250	23025251	23025252	23025253
Animal ID	No Treatment #1_D1	No Treatment #2_D1	No Treatment #3_D1	LIFE Biomaterial #1_D1	LIFE Biomaterial #2_D1	LIFE Biomaterial #3_D1	LIFE Biomaterial Anti-CD40 #1_D1	LIFE Biomaterial Anti-CD40 #2_D1
**HEART- The section of heart is a longitudinal section showing profiles of the right ventricle, left ventricle, and both atria.**	**N**	**N**	**N**	**N**	**N**	**N**	**N**	**N**
**LUNG - The section shows multiple anatomically normal lobes that are 75% inflated. There are occasional small foci of hemorrhage in the normal lung reportedly resulting from the euthanasia procedure.**	**N**	**N**	**N**	**N**	**N**	**N**	**N**	**N**
**SPLEEN - The spleen is architecturally correct, from a normal immunocompetent mouse strain with a 3:1 to 4:1 ratio of red pulp to white pulp. The red pulp contains robust extramedullary hematopoiesis, and the white pulp consists of numerous lymphoid follicles.**	**N**	**N**	**N**	**N**	**N**	**N**	**N**	**N**
**LIVER - There are two sections of liver lobe collected from non-fasted animals, that are anatomically normal.**	**N**				**N**			
Microgranulomas - Small aggregates of inflammatory cells, up to 100 cells, consisting of macrophages, lymphocytes, and neutrophils that often contain one or more entrapped degenerating hepatocytes. Mononuclear inflammatory cells predominate the cellular aggregates.			**1MF**	**1MF**				
Microabscesses - Small aggregates of inflammatory cells, up to 100 cells, consisting of macrophages, lymphocytes, and neutrophils that often contain one or more entrapped degenerating hepatocytes. Neutrophils are the predominate inflammatory cell in the cellular aggregates.		**2MF**				**1MF**	**2MF**	**3MF**
**KIDNEYS (LEFT AND RIGHT) - The kidneys are anatomically normal. There is a longitudinal profile of the left kidney and a cross-section of the right kidney. There is a normal piece of the adrenal gland at the pole of the left kidney in most slides.**	**N**	**N**	**N**	**N**	**N**	**N**	**N**	**N**

Scoring Definitions:

0= No finding 1= Minimal 2= Mild 3= Moderate 4= Marked 5= Severe
N= Normal M= Missing MF=Multifocal F=Focal D=Diffuse U=Unilateral
B=Bilateral

Mouse Accession	23025254
Animal ID	LIFE Biomaterial Anti-CD40 #3_D1
**HEART- The section of heart is a longitudinal section showing profiles of the right ventricle, left ventricle, and both atria.**	**N**
**LUNG - The section shows multiple anatomically normal lobes that are 75% inflated. There are occasional small foci of hemorrhage in the normal lung reportedly resulting from the euthanasia procedure.**	**N**
**SPLEEN - The spleen is architecturally correct, from a normal immunocompetent mouse strain with a 4:1 ratio of red pulp to white pulp. The red pulp contains robust extramedullary hematopoiesis, and the white pulp consists of numerous lymphoid follicles.**	**N**
**LIVER - Each slide contains two anatomically normal sections of liver lobe collected from non-fasted animals.**	
Microgranulomas - Small aggregates of inflammatory cells, up to 100 cells, consisting of macrophages, lymphocytes, and neutrophils that often contain one or more entrapped degenerating hepatocytes. Mononuclear inflammatory cells predominate the cellular aggregate.	
Periportal inflammatory infiltration - The perivascular and peribiliary connective tissue within portal triads is expanded by infiltration of mononuclear cells consisting of macrophages and lymphocytes.	**3MF**
Acute necrotizing hepatitis - Foci of inflammatory cells encompassing numerous necrotic hepatocytes and necrotic cellular debris, consisting of greater than 100 cells and greater than a millimeter in diameter. The primary inflammatory cell is the neutrophil.	**3MF**
Microabscesses - Small aggregates of inflammatory cells, up to 100 cells, consisting of macrophages, lymphocytes, and neutrophils that often contain one or more entrapped degenerating hepatocytes. Neutrophils are the predominate inflammatory cell in the cellular aggregate.	
**KIDNEYS (LEFT AND RIGHT) - The kidneys are anatomically normal. There is a longitudinal profile of the left kidney and a cross-section of the right kidney. There is a normal piece of the adrenal gland at the pole of the left kidney on most slides.**	**N**
Perivascular lymphocytic aggregate - At the corticomedullary junction in the renal cortex there are aggregates of lymphocytes that tend to expand the perivascular connective tissue space and minimally compress adjacent tissue.	

Table S2. Pathology report corresponding to day 1 post-treatment from harvested heart, lung, spleen, liver and kidneys tissues.

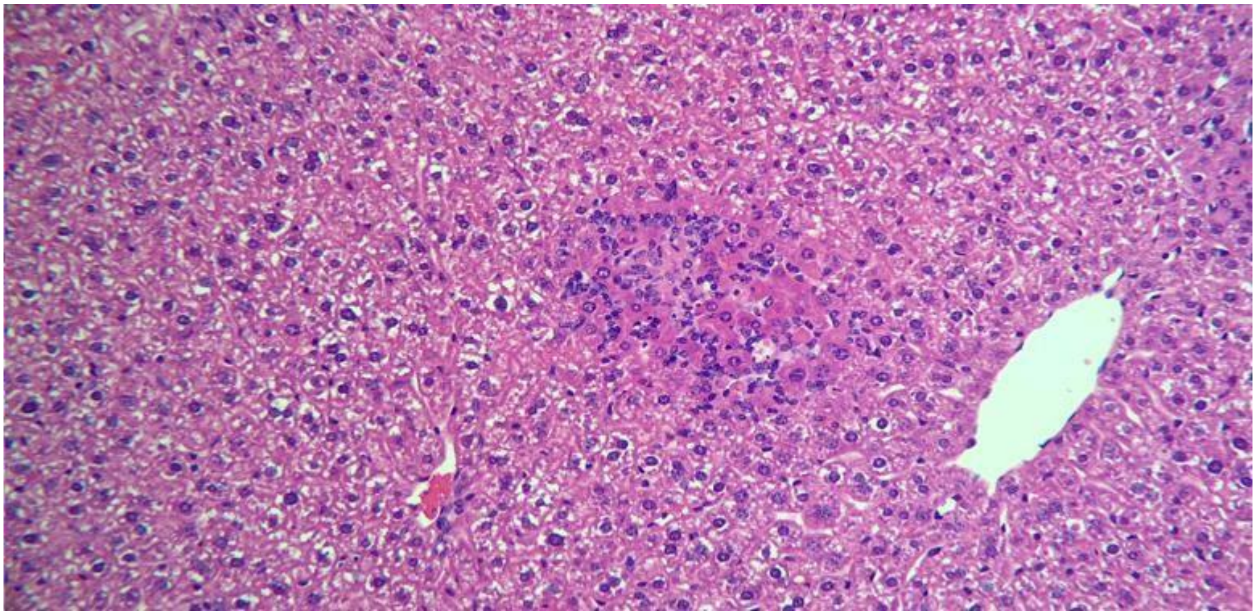

Mouse 23025252 liver with a microabscess typical of those reported in this group of mice. Note the large percentage of neutrophils in the inflammatory cell aggregate, which encompasses numerous degenerate and necrotic hepatocytes. 200X

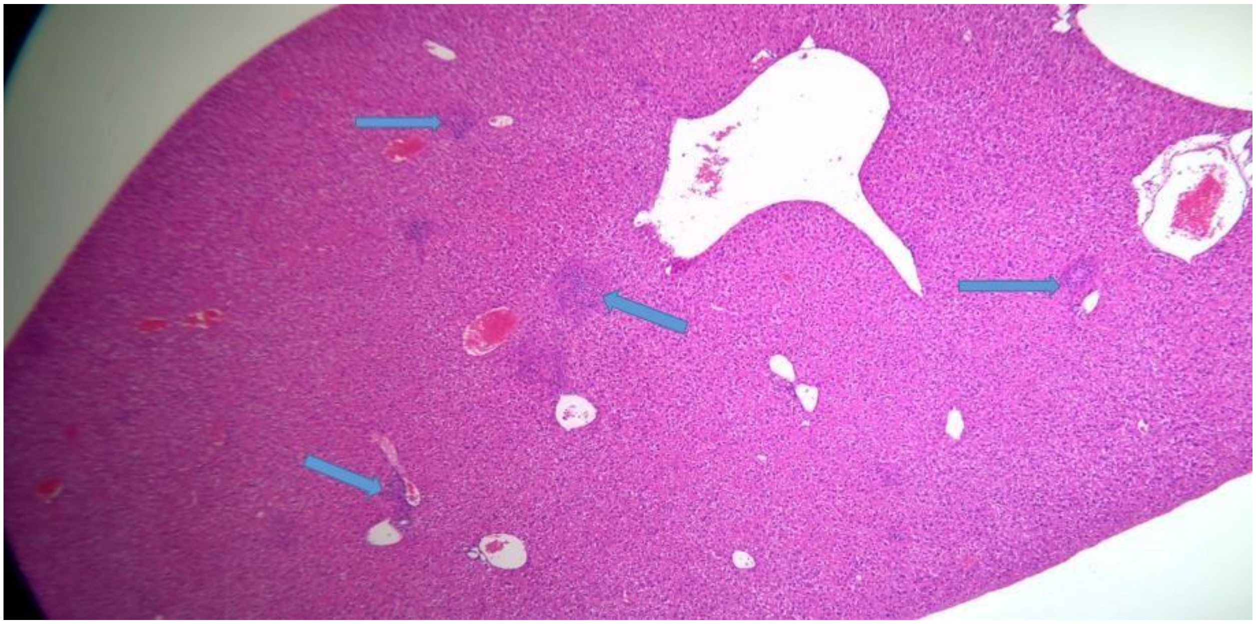

Mouse 23025254 liver with numerous (multifocal) foci of hepatocellular necrosis and admixed inflammatory cell infiltrates (arrows). 40X
