# Supplementary material for: Investigating the Use of a Liquid Immunogenic Fiducial Eluter Biomaterial in Cervical Cancer Treatment
Source: Cancers (Basel). 2024 Mar 20;16(6):1212. doi: 10.3390/cancers16061212 (PMC10969426; doi:10.3390/cancers16061212)
Supplement: Supplementary file 1 [file cancers-16-01212-s001.zip › Table S4.pdf]

VRL – Maryland, LLC
401 Professional Drive, Suite 210
Gaithersburg, MD 20879
Phone: 1-800-804-3586

Date: 10 November 2023
Client: Johns Hopkins University
Pathologist: Dr. Dan Ragland

Table S4. Histopathology Report for female mice (n = 3) 30-days post-treatment.

Accession #	23024684	23024685	23024686	23024687	23024688	23024689	23024690	23024691	23024692
**Mice ID#**	**No Treatment #1_D30**	**No Treatment #2_D30**	**No Treatment #3_D30**	**LIFE Biomaterial #1_D30**	**LIFE Biomaterial #2_D30**	**LIFE Biomaterial #3_D30**	**LIFE Biomaterial Anti-CD40 #1_D30**	**LIFE Biomaterial Anti-CD40 #2_D30**	**LIFE Biomaterial Anti-CD40 #3_D30**
**HEART**	N	N	N	N	N	N	N		N
Hemorrhage, alveolar and interstitial; often iatrogenic & associated with method of euthanasia at necropsy								1MF	
**LUNG**	N	N	N	N	N	N	N	N	N
**SPLEEN**	N	N	N	N	N	N	N	N	N
**LIVER**		N						N	N
Mirogranuloma: A small inflammatory focus typically less than 100 cells, consisting of mostly mononuclear cells (lymphocytes & macrophages) with fewer neutrophils and at least one encompassed degenerate hepatocyte and necrotic cellular debris	1MF		1MF		1MF	1MF	1MF		
Microabscess: A small inflammatory focus typically less than 100 cells, consisting of mostly neutrophils and lesser lymphocytes and or			1MF	2MF					

macrophages, encompassing one or more degenerate hepatocytes, and necrotic cellular debris									
Lymphocytic aggregate, perivascular, periportal: A small aggregate of lymphocytes deposited in the perivascular connective tissue adjacent to portal vein and/or the bile ducts.				1F			1F		
**KIDNEY**	**N**	**N**	**N**	**N**	**N**	**N**	**N**	**N**	**N**

Scoring Definitions:

0= No finding 1= Minimal 2= Mild 3= Moderate 4= Marked 5= Severe

N= Normal M= Missing MF=Multifocal F=Focal D=Diffuse U=Unilateral

B=Bilateral

Table S3. Pathology report corresponding to day 30 post-treatment from harvested heart, lung, spleen, liver and kidneys tissues.

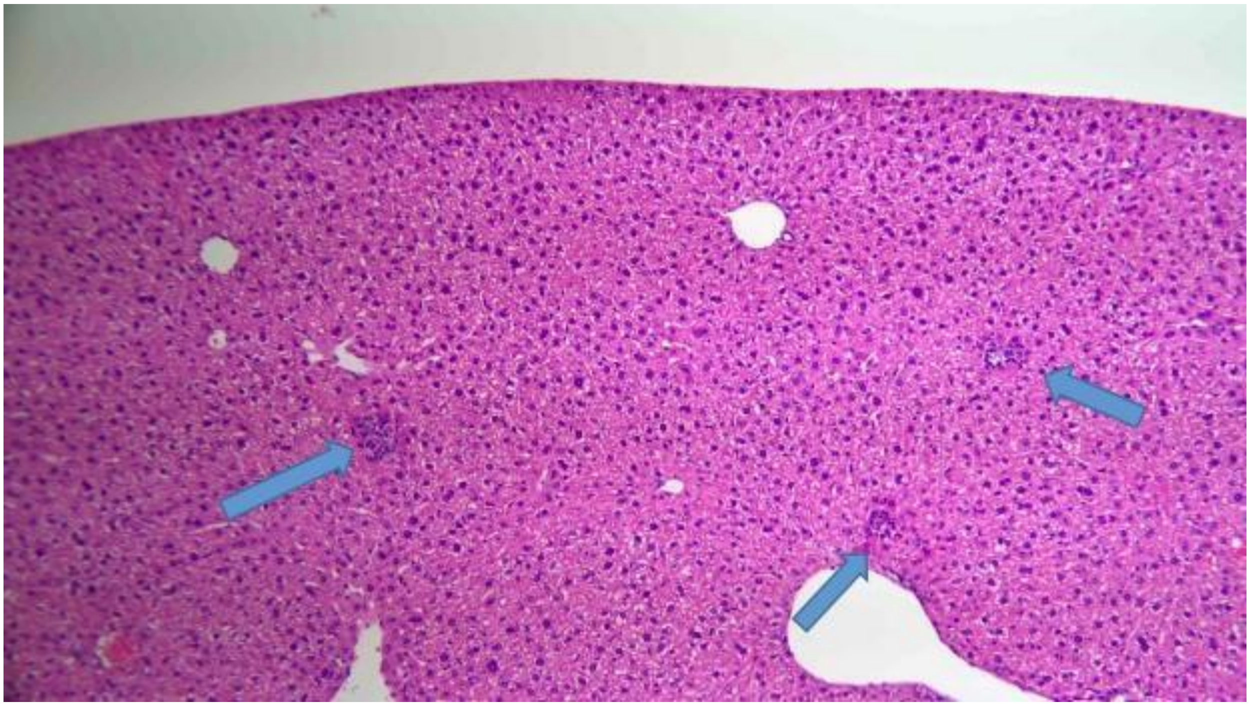

Mouse 23024689 liver showing three small aggregates of inflammatory cells (arrows) consisting of a mixture of lymphocytes, neutrophils, and macrophages, which often contain one or more degenerating hepatocytes (100X). The aggregates are classified as microgranulomas, or microabscesses, if the neutrophilic component is the predominant cell type. These small aggregates of inflammatory cells are typical of those seen in almost all of the mice submitted for evaluation.
