# Supplementary material for: Investigating the Use of a Liquid Immunogenic Fiducial Eluter Biomaterial in Cervical Cancer Treatment
Source: Cancers (Basel). 2024 Mar 20;16(6):1212. doi: 10.3390/cancers16061212 (PMC10969426; doi:10.3390/cancers16061212)
Supplement: Supplementary file 1 [file cancers-16-01212-s001.zip › Table S5.pdf]

VRL – Maryland, LLC
401 Professional Drive, Suite 210
Gaithersburg, MD 20879
Phone: 1-800-804-3586

Date: 04 December 2023
Client: Johns Hopkins University
Pathologist: Dr. Dan Ragland

Table S5. Histopathology Report for Female mice (n = 3) Day 60 post-treatment.

Mouse Accession	23027307	23027308	23027309	23027310	23027311	23027312	23027313	23027314	23027315
Animal ID	No Treatment_#1 _D60	No Treatment_#2 _D60	No Treatment_#3 _D60	LIFE Biomaterial #1 _D60	LIFE Biomaterial #2 _D60	LIFE Biomaterial #3 _D60	LIFE Biomaterial Anti-CD40 #1 _D60	LIFE Biomaterial Anti-CD40 #2 _D60	LIFE Biomaterial Anti-CD40 #3 _D60
**HEART- The section of heart is a longitudinal section showing profiles of the right ventricle, left ventricle, and both atria.**	N	N	N	N	N	N	N	N	N
**LUNG - The section shows multiple anatomically normal lobes that are 75% inflated. There are occasional small foci of hemorrhage in the normal lung reportedly resulting from the euthanasia procedure.**	N	N	N	N	N		N	N	N
Extensive area of pulmonary consolidation composed of mixed perivascular and peribronchiolar inflammatory infiltrates that expand into the adjacent alveollar lumens. The epithelial lining of the affected airways are piled up, are hypertrophic, and there are intraluminal histiocytes, neutrophils, lymphocytes, and hemorrahge.						3F			
**SPLEEN - The spleen is architecturally correct, from a normal immunocompetent mouse strain with a 4:1 ratio of red pulp to white pulp. The red pulp contains robust extramedullary hematopoiesis, and the white pulp consists of numerous lymphoid follicles.**	N	N	N	N	N	N	N	N	N
**LIVER - There are two sections of liver lobe collected from non-fasted animals, that are anatomically normal.**									
Microgranulomas - Small aggregates of inflammatory cells, up to 100 cells, consisting of macrophages, lymphocytes, and neutrophils that often contain one or more entrapped degenerating hepatocytes. In microgranulomas the mononuclear inflammatory cells predominate the cellular aggregate.			2MF		1MF			1MF	1MF
Microabscesses - Small aggregates of inflammatory cells, up to 100 cells, consisting of macrophages, lymphocytes, and neutrophils that often contain one or more entrapped degenerating hepatocytes. In microabscesses the neutrophils are the predominate inflammatory cell in the cellular aggregate.	1MF	2MF		2MF		2MF	1MF		
**KIDNEYS (LEFT AND RIGHT) - The kidneys are anatomically normal. There is also a section of normal adrenal gland on each slide.**	N	N	N		N	N		N	N
Renal Pelvis: Suburothelial, interstitial, and perivascular lymphocytic infiltrates.				1MF			1MF		

Scoring Definitions:

0= No finding 1= Minimal 2= Mild 3= Moderate 4= Marked 5= Severe
N= Normal M= Missing MF=Multifocal F=Focal D=Diffuse U=Unilateral
B=Bilateral

Table S4. Pathology report corresponding to day 60 post-treatment from harvested heart, lung, spleen, liver and kidneys tissues.

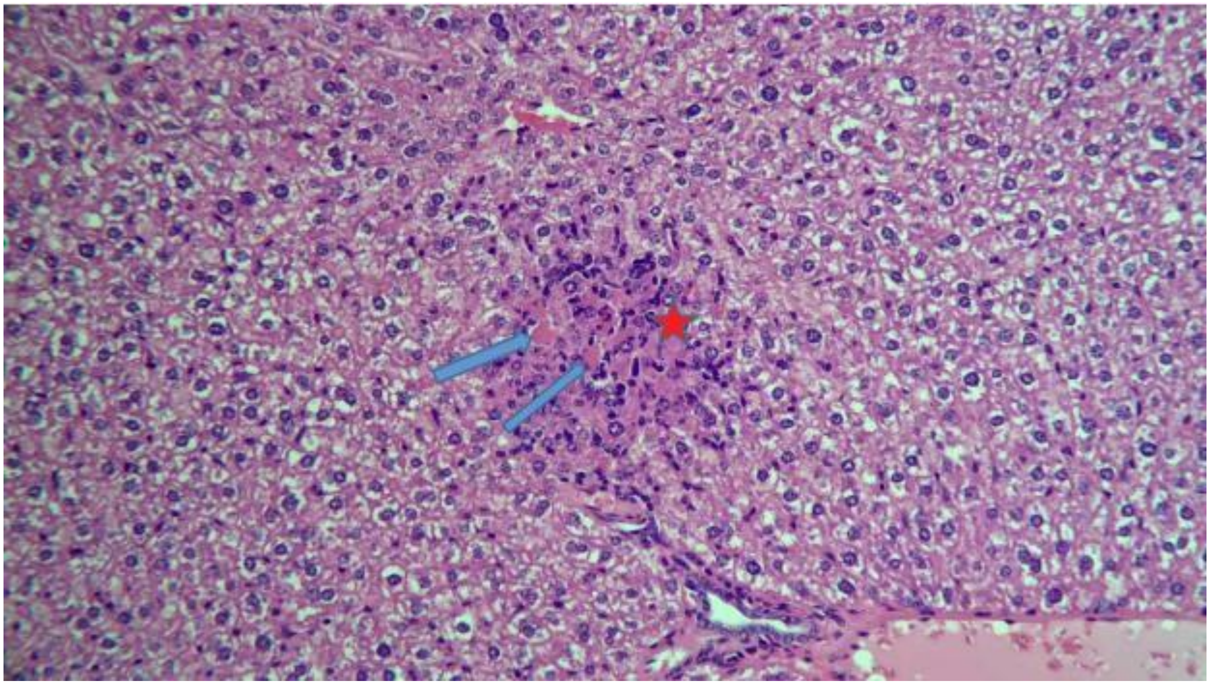

Mouse 23027314 liver showing an aggregate of inflammatory cells (star) consisting of a mixture of lymphocytes, neutrophils, and macrophages, which often contain one or more degenerating hepatocytes. 100X The aggregates are classified as microgranulomas if mononuclear inflammatory cells comprise the majority of the aggregates, and microabscesses if the neutrophilic component is the predominant cell type. These aggregates of inflammatory cells are typical of those seen in all of the mice submitted for evaluation. The only difference is in the size of the aggregates and their distribution.

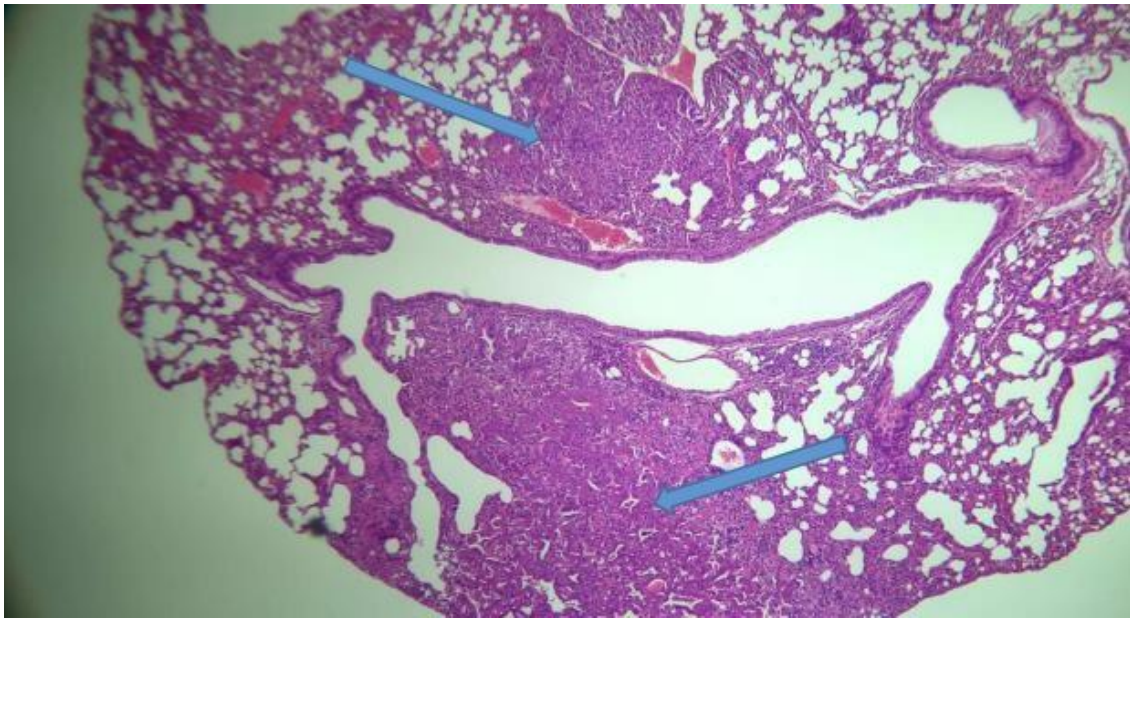

Mouse 23027312 lung with a consolidated area in which there is a mixed population of perivascular and peribronchiolar inflammatory cells. The alveolar lumens are filled with histiocytes, neutrophils, lymphocytes, and the alveolar epithelium is hyperplastic and hypertrophic. 40X
