# Supplementary material for: Investigating the Use of a Liquid Immunogenic Fiducial Eluter Biomaterial in Cervical Cancer Treatment
Source: Cancers (Basel). 2024 Mar 20;16(6):1212. doi: 10.3390/cancers16061212 (PMC10969426; doi:10.3390/cancers16061212)
Supplement: Supplementary file 1 [file cancers-16-01212-s001.zip › Table S6.pdf]

VRL – Maryland, LLC
401 Professional Drive, Suite 210
Gaithersburg, MD 20879
Phone: 1-800-804-3586

Date: 26 December 2023
Client: Johns Hopkins University
Pathologist: Dr. Dan Ragland

Table S6. Histopathology Report for Female mice (n = 3) Day 90 post-treatment.

Mouse Accession	23028887	23028888	23028889	23028890	23028891	23028892	23028893	23028894	2328895
Animal ID	No Treatment #1_D90	No Treatment #2_D90	No Treatment #3_D90	LIFE Biomaterial #1_D90	LIFE Biomaterial #2_D90	LIFE Biomaterial #3_D90	LIFE Biomaterial Anti-CD40 #1_D90	LIFE Biomaterial Anti-CD40 #2_D90	LIFE Biomaterial Anti-CD40 #3_D90
**HEART- The section of heart is a longitudinal section showing profiles of the right ventricle, left ventricle, and both atria.**	N	N	N	N	N	N	N	N	N
**LUNG - The section shows multiple anatomically normal lobes that are 75% inflated. There are occasional small foci of hemorrhage in the normal lung reportedly resulting from the euthanasia procedure.**	N	N	N	N	N	N	N	N	N
**SPLEEN - The spleen is architecturally correct, from a normal immunocompetent mouse strain with a 4:1 ratio of red pulp to white pulp. The red pulp contains robust extramedullary hematopoiesis, and the white pulp consists of numerous lymphoid follicles.**	N	N	N	N	N	N	N	N	N
**LIVER - There are two sections of liver lobe collected from non-fasted animals, that are anatomically normal.**		N							
Microgranulomas - Small aggregates of inflammatory cells, up to 100 cells, consisting of macrophages, lymphocytes, and neutrophils that often contain one or more entrapped degenerating hepatocytes. In microgranulomas the mononuclear inflammatory cells predominate the cellular aggregate.	1MF			1MF	1MF	1MF	1MF	1MF	1MF
Microabscesses - Small aggregates of inflammatory cells, up to 100 cells, consisting of macrophages, lymphocytes, and neutrophils that often contain one or more entrapped degenerating hepatocytes. In microabscesses the neutrophils are the predominate inflammatory cell in the cellular aggregate.			1MF						
**KIDNEYS (LEFT AND RIGHT) - The kidneys are anatomically normal. There is also a section of normal adrenal gland on each slide.**	N	N	N	N	N	N	N	N	N

Scoring Definitions:

0= No finding 1= Minimal 2= Mild 3= Moderate 4= Marked 5= Severe
N= Normal M= Missing MF=Multifocal F=Focal D=Diffuse U=Unilateral
B=Bilateral

Table S5. Pathology report corresponding to day 90 post-treatment from harvested heart, lung, spleen, liver and kidneys tissues.
