# Supplementary material for: Investigating the Use of a Liquid Immunogenic Fiducial Eluter Biomaterial in Cervical Cancer Treatment
Source: Cancers (Basel). 2024 Mar 20;16(6):1212. doi: 10.3390/cancers16061212 (PMC10969426; doi:10.3390/cancers16061212)
Supplement: Supplementary file 1 [file cancers-16-01212-s001.zip › Tables S1-S2.pdf]

Table S1. Hepatic and Renal function panels in female mice at different time points post-treatment (data presented as mean ± SD, n = 3).

Time point	Key Parameters	COHORTS OF FEMALE MICE		
		No Treatment	LIFE Biomaterial	LIFE Biomaterial_20 ug Anti-CD40
Day 1	**Direct Bilirubin** (0.00 – 0.54 mg/dL)	0.17 ± 0.09	0.16 ± 0.05	0.13 ± 0.05
	**Indirect Bilirubin**	0.05 ± 0.05	0.03 ± 0.05	0.10 ± 0.10
	**Total Bilirubin** (0.17 – 0.53 mg/dL)	0.22 ± 0.05	0.20 ± 0.00	0.20 ± 0.10
	**Creatinine** (0.12 – 0.43 mg/dL)	0.14 ± 0.01	0.13 ± 0.03	0.13 ± 0.03
	**BUN** (9.42 – 31.53 mg/dL)	23.50 ± 2.38	23.33 ± 0.57	19.67 ± 4.16
	**ALT** (15.00 – 80.10 U/L)	19.25 ± 3.09	12.00 ± 3.46	217.30 ± 116.76
	**AST** (33.95 – 268.47 U/L)	35.50 ± 2.08	30.33 ± 3.78	191.30 ± 77.10
	**Alkaline Phosphatase** (9.60 – 218.85 U/L)	151.80 ± 29.93	99.67 ± 4.93	76.33 ± 13.57
	**GGT** (3.79 – 11.62 mg/dL)	6.99 ± 0.00	6.99 ± 0.00	6.99 ± 0.00
	**Calcium** (8.28 – 12.27 mg/dL)	9.65 ± 0.17	10.43 ± 0.80	10.23 ± 0.15
	**Glucose** (106.74 – 353.74 mg/dL)	138.50 ± 38.30	129.30 ± 116.80	162.70 ± 21.19
	**Phosphorus** (3.61 – 13.19 mg/dL)	11.68 ± 1.71	9.70 ± 3.13	9.23 ± 0.41
	**Total Protein** (4.48 – 6.32 g/dL)	4.35 ± 0.12	4.36 ± 0.15	4.30 ± 0.10
	**Albumin** (1.92 – 4.11 g/dL)	2.97 ± 0.05	2.73 ± 0.05	2.86 ± 0.15
	**Globulin** (0.92 – 3.64 g/dL)	1.37 ± 0.15	1.63 ± 0.11	1.43 ± 0.23
	**Sodium** (124.81 – 160.31 mmol/L)	147.4 ± 0.55	146.60 ± 4.53	147.20 ± 2.21
	**Potassium** (2.17 – 8.18 mmol/L)	4.86 ± 0.16	4.82 ± 0.05	4.83 ± 0..16
	**Bicarbonate** (5.86 – 30.19 mEq/L)	12.83 ± 1.42	14.00 ± 2.05	12.30 ± 0.75
	**ALB/GLOB Ratio**	2.18 ± 0.26	1.68 ± 0.10	2.04 ± 0.39
	**BUN/Creatinine Ratio**	163.90 ± 23.52	179.20 ± 33.01	147.20 ± 12.94
	**Na/K Ratio**	30.33 ± 0.98	30.36 ± 0.67	30.48 ± 0.62
Day 30	**Direct Bilirubin** (0.00 – 0.54 mg/dL)	0.23 ± 0.12	0.16± 0.06	0.30 ± 0.14
	**Indirect Bilirubin**	0.10 ± 0.10	0.06 ± 0.06	0.10 ± 0.14
	**Total Bilirubin** (0.17 – 0.53 mg/dL)	0.33 ± 0.15	0.23 ± 0.06	0.4 ± 0.28
	**Creatinine** (0.12 – 0.43 mg/dL)	0.21± 0.07	0.23 ± 0.15	0.25 ± 0.01
	**BUN** (9.42 – 31.53 mg/dL)	24.33 ± 2.08	18.33 ± 0.58	17.50 ± 0.71
	**ALT** (15.00 – 80.10 U/L)	15.33 ± 6.11	13.00 ± 2.65	7.50 ± 2.12
	**AST** (33.95 – 268.47 U/L)	65.67± 45.35	40.67± 19.50	28.50 ± 2.12
	**Alkaline Phosphatase** (9.60 – 218.85 U/L)	110.40 ± 6.85	105.30 ± 4.93	99.50 ± 16.26
	**GGT** (3.79 – 11.62 mg/dL)	6.99 ± 0.00	7.32 ± 0.58	6.99 ± 0.01
	**Calcium** (8.28 – 12.27 mg/dL)	90.33 ± 1.32	9.43 ± 0.05	9.70 ± 0.28
	**Glucose** (106.74 – 353.74 mg/dL)	266.30 ± 45.08	258.30 ± 23.54	241.50 ± 17.68
	**Phosphorus** (3.61 – 13.19 mg/dL)	8.93 ± 1.12	11.00 ± 3.36	13.85 ± 0.35
	**Total Protein** (4.48 – 6.32 g/dL)	4.60 ± 0.15	4.66 ± 0.06	4.55 ± 0.28
	**Albumin** (1.92 – 4.11 g/dL)	2.66 ± 0.40	2.90 ± 0.17	2.80 ± 0.14
	**Globulin** (0.92 – 3.64 g/dL)	1.93 ± 0.58	1.76 ± 0.15	1.75 ± 0.07
	**Sodium** (124.81 – 160.31 mmol/L)	150.80 ± 2.35	151.70 ± 2.25	148.80 ± 3.96
	**Potassium** (2.17 – 8.18 mmol/L)	4.40 ± 0.32	4.50± 0.17	4.63 ± 0.30
	**Bicarbonate** (5.86 – 30.19 mEq/L)	13.90 ± 3.28	12.73 ± 2.11	13.05 ± 0.64
	**ALB/GLOB Ratio**	1.49 ± 0.55	1.64 ± 0.15	1.65 ± 0.15

	**BUN/Creatinine Ratio**	128.10 ± 56.18	104.40 ± 68.20	70.19 ± 6.80
	**Na/K Ratio**	34.35 ± 2.62	33.71 ± 0.74	32.15 ± 1.25
**Day 60**	**Direct Bilirubin (**0.00 – 0.54 mg/dL)	0.16 ± 0.05	0.13 ± 0.05	0.23 ± 0.05
	**Indirect Bilirubin**	0.06 ± 0.05	0.03 ± 0.05	1.43 ± 0.00
	**Total Bilirubin (**0.17 – 0.53 mg/dL)	0.23 ± 0.05	0.16 ± 0.05	0.23 ± 0.05
	**Creatinine (**0.12 – 0.43 mg/dL)	0.27 ± 0.14	0.19 ± 0.10	0.22 ± 0.10
	**BUN (**9.42 – 31.53 mg/dL)	20.33 ± 2.52	24.33 ± 4.51	22.67 ± 4.51
	**ALT** (15.00 – 80.10 U/L)	17.0 ± 5.00	17.33 ± 4.73	25.00 ± 14.73
	**AST** (33.95 – 268.47 U/L)	36.00 ± 6.56	34.00 ± 4.58	44.00 ± 10.82
	**Alkaline Phosphatase** (9.60 – 218.85 U/L)	93.67± 5.13	106.3 ± 13.80	97.67± 10.21
	**GGT** (3.79 – 11.62 mg/dL)	6.99 ± 0.00	6.99 ± 0.00	6.99 ± 0.00
	**Calcium** (8.28 – 12.27 mg/dL)	9.66 ± 0.25	9.40 ± 0.20	9.60 ± 0.17
	**Glucose** (106.74 – 353.74 mg/dL)	160.± 84.90	221.70 ± 30.90	205.70 ± 12.06
	**Phosphorus** (3.61 – 13.19 mg/dL)	10.30 ± 3.85	7.00 ± 1.47	9.03 ± 1.27
	**Total Protein** (4.48 – 6.32 g/dL)	4.60 ± 0.10	4.60 ± 0.26	4.36 ± 0.06
	**Albumin** (1.92 – 4.11 g/dL)	2.86 ± 0.00	2.86 ± 0.00	2.80 ± 0.00
	**Globulin** (0.92 – 3.64 g/dL)	1.73 ± 0.15	1.73 ± 0.15	1.56 ± 0.15
	**Sodium** (124.81 – 160.31 mmol/L)	145.1± 1.15	143.6 ± 1.00	145.1 ± 4.03
	**Potassium** (2.17 – 8.18 mmol/L)	4.21± 0.09	4.18 ± 0.12	4.24 ± 0.26
	**Bicarbonate** (5.86 – 30.19 mEq/L)	15.77± 1.32	16.17± 2.16	14.00 ± 2.20
	**ALB/GLOB Ratio**	1.63 ± 0.17	1.60 ± 0.09	1.80 ± 0.31
	**BUN/Creatinine Ratio**	0.50 ± 79.30	159.70 ± 115.72	115.30 ± 42.83
	**Na/K Ratio**	34.44± 0.49	34.31 ± 1.01	34.24 ± 1.24
**Day 90**	**Direct Bilirubin (**0.00 – 0.54 mg/dL)	0.30 ± 0.14	0.13 ± 0.06	0.20 ± 0.10
	**Indirect Bilirubin**	0.05 ± 0.07	0.06 ± 0.06	0.03 ± 0.05
	**Total Bilirubin (**0.17 – 0.53 mg/dL)	0.35 ± 0.21	0.20 ± 0.00	0.23 ± 0.05
	**Creatinine (**0.12 – 0.43 mg/dL)	0.20 ± 0.11	0.32 ± 0.03	0.21 ± 0.05
	**BUN (**9.42 – 31.53 mg/dL)	24.50 ± 2.12	24.00 ± 2.64	26.33 ± 1.52
	**ALT** (15.00 – 80.10 U/L)	26.00 ± 8.49	25.67 ± 3.06	20.67± 4.61
	**AST** (33.95 – 268.47 U/L)	58.00 ± 15.55	46.67± 3.06	37.67 ± 5.03
	**Alkaline Phosphatase** (9.60 – 218.85 U/L)	75.50 ± 4.95	88.03 ± 17.57	89.67±12.22
	**GGT** (3.79 – 11.62 mg/dL)	6.99 ± 0.00	6.99 ± 0.00	7.66 ± 1.16
	**Calcium** (8.28 – 12.27 mg/dL)	9.50 ± 0.14	9.83 ± 0.06	9.43 ± 0.15
	**Glucose** (106.74 – 353.74 mg/dL)	226.50 ± 38.39	201.70 ± 52.79	243.00 ± 29.46
	**Phosphorus** (3.61 – 13.19 mg/dL)	9.25 ± 2.47	9.70 ± 1.13	8.80 ± 1.63
	**Total Protein** (4.48 – 6.32 g/dL)	4.80 ± 0.57	4.76 ± 0.32	4.53 ± 0.23
	**Albumin** (1.92 – 4.11 g/dL)	2.90 ± 0.14	3.03 ± 0.11	2.50 ± 0.52
	**Globulin** (0.92 – 3.64 g/dL)	1.90 ± 0.42	1.73 ± 0.38	2.03 ± 0.50
	**Sodium** (124.81 – 160.31 mmol/L)	147.50 ± 4.03	145.30 ± 1.96	149.80 ± 1.78
	**Potassium** (2.17 – 8.18 mmol/L)	4.23 ± 0.21	4.00 ± 0.16	4.11 ± 0.14
	**Bicarbonate** (5.86 – 30.19 mEq/L)	13.10 ± 3.25	13.93 ± 1.14	14.60 ±1.64
	**ALB/GLOB Ratio**	1.55 ± 0.28	1.82 ± 0.49	1.32 ± 0.59
	**BUN/Creatinine Ratio**	141.10 ± 83.33	73.86 ± 9.52	130.50 ± 31.56
	**Na/K Ratio**	34.84 ± 0.73	36.30 ± 0.92	36.42 ± 0.88

Table S1. Following the vena cava blood sampling method, blood sera were collected from female mice to evaluate different panels of Hepatic and Renal functions corresponding to days 1, 30, 60 and 90 post-treatment with the annotated reference range for each parameter as noted in the table. Alanine Transaminase (ALT),

Aspartate aminotransferase (AST), Gamma-glutamyl transferase (GGT), Blood Urea Nitrogen (BUN), Albumin/Globulin (ALB/GLOB), Sodium/Potassium (Na/K).

Table S2. Hematology analysis from female mice at different time points post-treatment (data presented as mean ± SD, *n* = *3*).

Time point	Key Parameters	COHORTS OF FEMALE MICE		
		No Treatment	LIFE Biomaterial	LIFE Biomaterial_20ug Anti-CD40
Day 1	**WBC** (3.20 – 12.70 K/uL)	3.50 ± 0.31	2.83 ± 0.59	4.03 ± 1.01
	**RBC** (7.00 – 10.10 M/uL)	8.41 ± 0.23	7.79 ± 0.38	8.40 ± 0.45
	**HGB** (11.80 – 14.90 g/dL)	14.05 ± 0.17	13.00 ± 0.52	13.60 ± 0.80
	**MCH** (13.80 – 18.40 pg)	15.60 ± 0.55	15.80 ± 0.45	15.73 ± 0.15
	**MCHC** (31.00 – 34.70 g/dL)	16.68 ± 1.04	16.67 ± 0.98	16.17 ± 0.25
	**MCV** (42.20 – 59.20 fL)	54.08 ± 1.40	54.00 ± 2.16	52.63 ± 0.28
	**MPV**	10.00 ± 2.27	10.97 ± 2.43	10.17 ± 2.80
	**CH**	13.93 ± 0.12	14.03 ± 0.25	13.70 ± 0.26
	**CHCM**	25.78 ± 0.86	26.30 ± 0.80	26.10 ± 0.51
	**HDW**	1.73 ± 0.05	1.74 ± 0.10	1.79 ± 0.12
	**PLT** (766.0 – 1657.0 K/uL)	704.80 ± 296.12	530.30 ± 406.36	688.30 ± 114.78
	**LYMPH COUNT** (3.80 – 8.90 K/uL)	3.04 ± 0.35	3.55 ± 0.29	1.25 ± 0.62
	**LYMPH %**	86.65± 2.50	76.47 ± 4.84	56.43 ± 4.75
	**NEUT COUNT** (0.50 – 2.00 K/uL)	0.28 ± 0.00	0.77 ± 0.31	0.6 ± 0.30
	**NEUT %**	7.95 ± 0.86	16.20 ± 4.68	29.00 ± 0.86
	**MONO COUNT** (0.00 – 0.30 K/uL)	0.04 ± 0.01	0.04 ± 0.01	0.05 ± 0.04
	**MONO %**	1.37 ± 0.54	0.86 ± 0.25	2.26 ± 1.07
	**EOS COUNT** (0.00 – 0.40 K/uL)	0.09 ± 0.03	0.09 ± 0.04	0.12 ± 0.04
	**EOS %**	2.85 ± 1.15	3.90 ± 0.51	7.30 ± 3.13
	**BASO COUNT** (0.00 – 0.10 K/uL)	0.00± 0.00	0.01 ± 0.00	0.01 ± 0.00
	**BASO %**	0.10 ± 0.00	0.13 ± 0.05	0.63 ± 0.49
	**LUC COUNT**	0.03 ± 0.01	0.11 ± 0.04	0.09 ± 0.02
	**LUC %**	1.05 ± 0.28	2.43 ± 0.83	4.43 ± 1.26
	**RDW %** (11.70 – 15.10)	12.78 ± 0.29	13.00 ± 0.55	12.57 ± 0.28
	**HCT %** (36.70 – 46.80)<br>**RETICS %**	45.50 ± 1.45<br>3.75 ± 0.35	42.03 ± 0.46<br>4..03 ± 1.00	44.35 ± 2.61<br>3.44 ± 0.74
Day 30	**WBC** (3.20 – 12.70 K/uL)	2.19 ± 1.89	1.32 ± 0.10	3.48 ± 1.11
	**RBC** (7.00 – 10.10 M/uL)	8.53 ± 0.10	8.21 ± 0.28	8.31± 0.34
	**HGB** (11.80 – 14.90 g/dL)	14.03 ± 0.21	13.50 ± 0.28	13.65± 0.49

	**MCH** (13.80 – 18.40 pg)	16.47 ± 0.12	16.40 ± 0.14	16.45 ±0.07
	**MCHC** (31.00 – 34.70 g/dL)	31.80 ± 0.44	30.65 ± 0.49	31.50 ± 1.41
	**MCV** (42.20 – 59.20 fL)	53.30 ± 0.95	51.60 ± 0.35	51.45 ± 2.62
	**MPV**	11.77± 3.13	9.9 ± 2.69	8.45 ± 0.49
	**CH**	13.67± 0.15	13.55± 0.07	13.70 ± 0.00
	**CHCM**	26.43± 0.67	25.25 ± 0.07	26.30 ± 1.41
	**HDW**	1.78 ± 0.06	1.71 ± 0.02	1.77± 0.11
	**PLT** (766.0 – 1657.0 K/uL)	624.00 ± 292.11	737.00 ± 355.67	1058.00 ± 95.46
	**LYMPH COUNT** (3.80 – 8.90 K/uL)	1.73 ± 1.50	1.13 ± 0.22	2.91± 0.72
	**LYMPH %**	79.07± 1.53	85.6 ± 2.61	84.65 ± 6.43
	**NEUT COUNT** (0.50 – 2.00 K/uL)	0.28 ± 0.28	0.11 ± 0.02	0.38± 0.27
	**NEUT %**	12.10 ± 2.27	8.85 ± 2.05	10.10 ± 4.53
	**MONO COUNT** (0.00 – 0.30 K/uL)	0.03 ± 0.03	0.01 ± 0.00	0.03± 0.02
	**MONO %**	1.73 ± 0.15	0.70 ± 0.14	1.05± 0.21
	**EOS COUNT** (0.00 – 0.40 K/uL)	0.10 ± 0.03	0.11 ± 0.01	0.20± 0.06
	**EOS %**	0.06 ± 1.06	0.03 ± 0.64	0.07± 0.00
	**BASO COUNT** (0.00 – 0.10 K/uL)	0.01 ± 0.00	0 .0 ± 0.00	0.01± 0.00
	**BASO %**	0.40 ± 0.10	0.15 ± 0.21	0.40 ± 0.00
	**LUC COUNT**	0.70 ± 0.05	0.02 ± 0.01	0.07± 0.04
	**LUC %**	3.43 ± 1.02	1.90 ± 0.28	1.85± 0.64
	**RDW %** (11.70 – 15.10)	12.63 ± 0.21	13.00± 0.42	12.95± 0.35
	**HCT %** (36.70 – 46.80)	44.10 ± 1.13	44.00 ± 1.70	43.45± 0.35
	**RETICS %**	2.89 ± 0.51	3.50± 0.06	3.08± 0.25
**Day 60**	**WBC** (3.20 – 12.70 K/uL)	5.45 ± 1.03	4.14 ± 2.72	5.04 ± 2.17
	**RBC** (7.00 – 10.10 M/uL)	8.68 ± 0.45	8.72 ± 0.06	8.74 ± 0.23
	**HGB** (11.80 – 14.90 g/dL)	14.20 ± 0.14	14.05 ± 0.21	13.95 ± 0.21
	**MCH** (13.80 – 18.40 pg)	16.35 ± 0.64	16.1 ± 0.14	16.00± 0.14
	**MCHC** (31.00 – 34.70 g/dL)	30.75 ± 0.78	31.15 ± 0.78	31.05 ± 0.78
	**MCV** (42.20 – 59.20 fL)	53.30 ± 3.39	51.60 ± 0.42	51.45 ± 0.64
	**MPV**	10.30 ± 0.14	10.55 ± 0.92	8.80 ± 0.71
	**CH**	14.00 ± 0.42	13.65 ± 0.21	13.45 ± 0.07
	**CHCM**	26.35 ± 0.78	26.50± 0.57	26.20 ± 0.42
	**HDW**	1.76 ± 0.01	1.87± 0.07	1.78 ± 0.06
	**PLT** (766.0 – 1657.0 K/uL)	545.50 ± 50.20	687.50 ± 109.60	917.50 ± 105.36
	**LYMPH COUNT** (3.80 – 8.90 K/uL)	4.73 ± 0.83	3.58 ± 2.57	4.36 ± 1.80
	**LYMPH %**	86.85 ± 1.20	84.30 ± 6.65	86.95 ± 1.77
	**NEUT COUNT** (0.50 – 2.00 K/uL)	0.46 ± 0.11	0.33 ± 0.05	0.35 ± 0.18
	**NEUT %**	8.40 ± 0.57	9.55 ± 5.16	6.75 ± 0.64
	**MONO COUNT** (0.00 – 0.30 K/uL)	0.07± 0.01	0.06 ± 0.00	0.04 ± 0.04
	**MONO %**	0.07± 0.21	0.06 ± 1.06	0.045 ± 0.35
	**EOS COUNT** (0.00 – 0.40 K/uL)	0.10 ± 0.04	0.11 ± 0.04	0.20 ± 0.11
	**EOS %**	1.90 ± 0.57	3.05 ± 0.78	3.80 ± 0.71

	**BASO COUNT** (0.00 – 0.10 K/uL)	0.03 ± 0.02	0.02 ± 0.02	0.00 ± 0.00
	**BASO %**	0.4 ± 0.35	0.45 ± 0.35	0.10 ± 0.00
	**LUC COUNT**	0.05 ± 0.00	0.04 ± 0.02	0.08 ± 0.04
	**LUC %**	1.00 ± 0.00	0.900 ± 0.00	1.55 ± 0.07
	**RDW %** (11.70 – 15.10)	13.20 ± 0.71	13.55 ± 0.92	13.05 ± 0.07
	**HCT %** (36.70 – 46.80)	46.20 ± 0.57	45.10 ± 0.71	44.95 ± 0.64
	**RETICS %**	3.68 ± 1.42	3.81± 0.58	2.77± 0.59
**Day 90**	**WBC** (3.20 – 12.70 K/uL)	3.72 ± 0.56	2.83 ± 0.83	4.03 ± 1.02
	**RBC** (7.00 – 10.10 M/uL)	8.95 ± 0.09	8.45 ± 0.88	8.67 ± 0.30
	**HGB** (11.80 – 14.90 g/dL)	13.95 ± 0.21	13.33 ± 1.27	13.63 ± 0.59
	**MCH** (13.80 – 18.40 pg)	15.60 ± 0.14	15.80 ± 0.26	15.73 ± 0.31
	**MCHC** (31.00 – 34.70 g/dL)	30.70 ± 0.00	30.90 ± 0.44	30.83 ± 0.25
	**MCV** (42.20 – 59.20 fL)	50.75 ± 0.35	51.07 ± 0.90	51.07 ± 0.68
	**MPV**	7.70 ± 0.28	9.86 ± 4.09	8.03 ± 0.85
	**CH**	13.75 ± 0.07	13.57 ± 0.06	13.60 ± 0.10
	**CHCM**	27.20 ± 0.14	26.60 ± 0.44	26.70 ± 0.17
	**HDW**	1.64 ± 0.07	1.63 ± 0.49	1.61 ± 0.25
	**PLT** (766.0 – 1657.0 K/uL)	396.00 ± 32.53	347.70 ± 456.56	727.00 ± 371.04
	**LYMPH COUNT** (3.80 – 8.90 K/uL)	3.22 ± 0.52	2.38 ± 0.73	3.45 ± 0.84
	**LYMPH %**	86.50 ± 0.85	84.10 ± 2.59	85.73 ± 0.93
	**NEUT COUNT** (0.50 – 2.00 K/uL)	0.26 ± 0.05	0.27 ± 0.10	0.34 ± 0.10
	**NEUT %**	7.05 ± 0.35	9.33 ± 2.05	8.60 ± 0.52
	**MONO COUNT** (0.00 – 0.30 K/uL)	0.07 ± 0.00	0.05 ± 0.02	0.04 ± 0.02
	**MONO %**	1.85 ± 0.21	1.93 ± 0.83	1.03 ± 0.23
	**EOS COUNT** (0.00 – 0.40 K/uL)	0.08± 0.01	0.09 ± 0.01	0.12 ± 0.04
	**EOS %**	2.10 ± 0.14	3.43 ± 1.00	3.06 ± 0.47
	**BASO COUNT** (0.00 – 0.10 K/uL)	0.00± 0.00	0.01 ± 0.01	0.01 ± 0.01
	**BASO %**	0.05± 0.07	0.16 ± 0.15	0.13 ± 0.06
	**LUC COUNT**	0.09 ± 0.01	0.02 ± 0.00	0.06 ± 0.03
	**LUC %**	2.40 ± 0.70	1.00 ± 0.17	1.46 ± 0.41
	**RDW %** (11.70 – 15.10)	12.85 ± 0.07	12.87± 0.49	12.67± 0.25
	**HCT %** (36.70 – 46.80)	45.23 ± 0.78	43.67± 4.61	43.35 ± 1.73
	**RETICS %**	4.16 ± 0.50	4.34 ± 1.25	3.49 ± 0.42

Table S2. Hematology analysis from whole blood samples collected from female mice at different days post-treatment (Days 1, 30, 60 and 90). Complete blood cell count parameters are measured and each parameter's reference range are mentioned accordingly. WBC: White Blood Cells; RBC: Red Blood Cells; HGB: Hemoglobin; MCH: Mean Corpuscular Hemoglobin; MCHC: Mean Corpuscular Hemoglobin Concentration; MCV: Mean Corpuscular Volume; PLT: Platelets; LYMPH COUNT: Lymphocytes Count; NEUT COUNT: Neutrophil Count; MONO COUNT: Monocyte Count; EOS COUNT: Eosinophil Count; BASO COUNT: basophil count; RDW%: Red Blood Cell width; HCT%: Hematocrit.
